# miR-30a inhibits endothelin A receptor and chemoresistance in ovarian carcinoma

**DOI:** 10.18632/oncotarget.6546

**Published:** 2015-12-10

**Authors:** Rosanna Sestito, Roberta Cianfrocca, Laura Rosanò, Piera Tocci, Elisa Semprucci, Valeriana Di Castro, Valentina Caprara, Gabriella Ferrandina, Andrea Sacconi, Giovanni Blandino, Anna Bagnato

**Affiliations:** ^1^ Translational Research Functional Departmental Area, Regina Elena National Cancer Institute, Rome, Italy; ^2^ Gynecologic Oncology Unit, Catholic University of Rome, Rome, Italy; ^3^ Translational Oncogenomic Unit, Regina Elena National Cancer Institute, Rome, Italy

**Keywords:** ovarian carcinoma, endothelin A receptor, miR-30a, chemoresistance, endothelin-1

## Abstract

Drug resistance remains the major clinical barrier to successful treatment in epithelial ovarian carcinoma (EOC) patients, and the evidence of microRNA involvement in drug resistance has been recently emerging. Endothelin-1 (ET-1)/ET_A_ receptor (ET_A_R) axis is aberrantly activated in chemoresistant EOC cells and elicits pleiotropic effects promoting epithelial-to-mesenchymal transition (EMT) and the acquisition of chemoresistance. However, the relationship between ET_A_R and miRNA is still unknown. Hence, in this study we evaluated whether dysregulation of miRNA might enhance ET_A_R expression in sensitive and resistant EOC cells. Based on bioinformatic tools, we selected putative miRNA able to recognize the 3′UTR of ET_A_R. An inverse correlation was observed between the expression levels of miR-30a and ET_A_R in both EOC cell lines and tumor samples. miR-30a was found to specifically bind to the 3′UTR of ET_A_R mRNA, indicating that ET_A_R is a direct target of miR-30a. Overexpression of miR-30a decreased Akt and mitogen activated protein kinase signaling pathway activation, cell proliferation, invasion, plasticity, EMT marker levels, and vascular endothelial growth factor release. Interestingly, ectopic expression of miR-30a re-sensitized platinum-resistant EOC cells to cisplatinum-induced apoptosis. Consistently, resistant EOC xenografts overexpressing miR-30a resulted in significantly less tumor growth than controls. Together our study provides a new perspective on the regulatory mechanism of ET_A_R gene. Interestingly, our findings highlight that blockade of ET_A_R regulatory axis is the mechanism underlying the tumor suppressor function of miR-30a in chemoresistant EOC cells.

## INTRODUCTION

Epithelial ovarian carcinoma (EOC) accounts for the highest tumor-related mortality in women with gynecologic malignancy [1]. The current therapy for advanced ovarian cancer is debulking surgery followed by chemotherapy containing cisplatinum agent and paclitaxel with the eventual addition of bevacizumab; however, successful long-term treatment is prevented by the development of drug resistance [2]. The identification of the molecular mechanisms underlying chemoresistance is mandatory to achieve advancement in EOC therapy. An aberrant activation of endothelin-1 (ET-1) axis, which consists of the ligand ET-1 and its G-protein coupled receptors (GPCR) endothelin A receptor (ET_A_R) and ET_B_R, is now recognized as a common mechanism underlying the progression of various solid tumors, including EOC [3]. In particular, ET_A_R overexpression is associated with the acquisition of chemoresistance and epithelial-to-mesenchymal transition (EMT) phenotype of EOC [3–7]. The ET-1/ET_A_R interaction triggers the activation of the survival pathways, such as phosphoinositide-3-kinase (PI-3K)/Akt and mitogen activated protein kinase (MAPK), thus protecting EOC cells from drug-induced apoptosis [4]. All these observations suggest that ET_A_R expression may be a predictor of chemoresistance, and that targeting ET_A_R could increase the sensitivity of EOC tumors to therapeutic agents. In this regard, we recently provide evidence that ET_A_R/β-arrestin-1 cooperates with Wnt signaling to acquire a chemoresistant phenotype through the amplification of ET-1 autocrine loop, thus sustaining EMT, stemness features, cell invasion and metastasis [8]. In addition to ET_A_R, ET_B_R also appears to have important regulatory roles in angiogenesis, lymphangiogenesis and evasion of the immune response [3, 8–14], indicating that ET_A_R and ET_B_R could serve as key targets for EOC. The dual receptor antagonist macitentan, interfering with ET_A_R, and microenvironment-associated elements expressing ET_B_R [12–15], inhibited growth, vascularization, intravasation and progression in EOC xenografts [8].

microRNA (miRNA) are small non-coding RNAs 19-25 nucleotides in length. Mature forms of miRNA silence gene expression by binding to the 3′ UTR of target mRNA and initiate translational repression or cleavage of cognate mRNA [16]. These molecules have been recognized as critical regulators of tumor development and progression, including EOC [17–20]. Moreover, recent studies demonstrated the involvement of miRNA in chemoresistance [21–24]. Despite the growing interest around miRNA in tumor progression and chemoresistance, currently there are no studies to clarify physiologic and pathologic implications on ET_A_R regulation by miRNA. In this study, we aimed to identify miRNA able to control ET_A_R expression overcoming therapy resistance in EOC cells. miR-30a has attracted much attention due to its important role in various biological and pathological processes, including EMT and tumor progression [25, 26]. However, whether miR-30a is involved in the chemoresistance onset of EOC and the underlying mechanisms remain poorly understood. Employing the Cancer Genome Atlas (TCGA) data [27], we found that miR-30a downregulation in high-grade serous ovarian cancer (HG-SOC), which reprents a large majority of EOC cases, is downregulated compared to normal tissue and is associated with poor prognosis. Moreover, we show that miR-30a is downregulated in chemoresistant EOC cells. Functional studies demonstrate that overexpression of miR-30a decreases cellular vitality, invasion, plasticity and EMT. ET_A_R is identified as a direct target of miR-30a, and their expression is inversely correlated in EOC cell lines and human tissue samples. Upregulation of miR-30a re-sensitizes resistant EOC cells to cisplatinum by binding ET_A_R. Overexpression of miR-30a inhibits tumor growth in cisplatinum-resistant xenografts. Our findings provide a new perspective on the regulatory mechanism of endogenous ET_A_R gene at the post-transcriptional level. Moreover, this study suggests that miR-30a/ET_A_R axis regulates EOC chemoresistance onset and that interfering with ET_A_R function could serve as an important therapeutic approach for EOC.

## RESULTS

### miR-30a is downregulated in chemoresistant ovarian cancer cells

**Figure 1:**
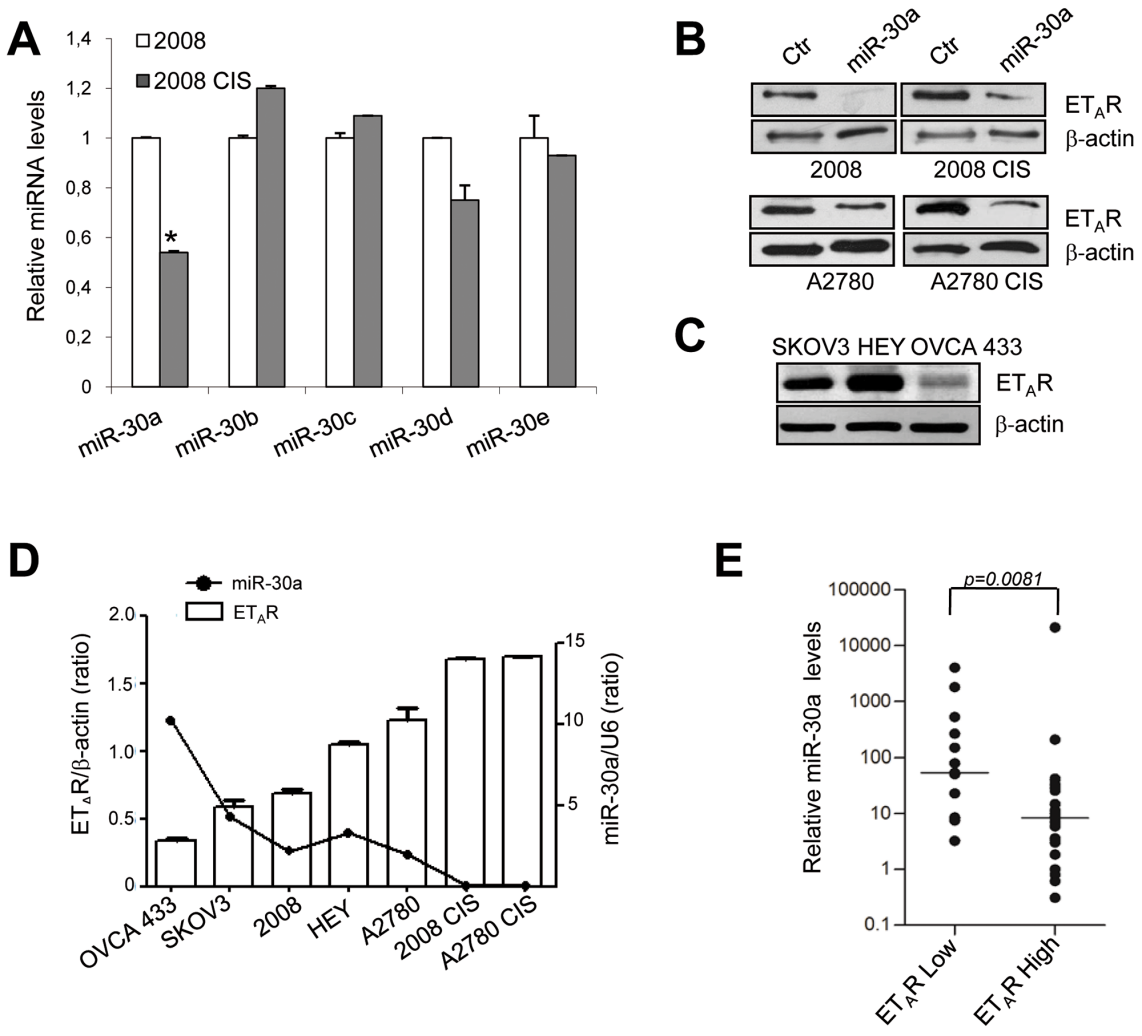
miR-30a is downregulated in chemoresistant EOC cells **A.** Expression of miR-30 family members in sensitive and resistant 2008 cells measured by qPCR. miRNA levels are normalized using endogenous U6 snRNA. Values are the mean ± SD (*n* = 3; *, *p* < 0.005 compared to sensitive cells). Lysates from sensitive and resistant 2008 and A2780 cells transfected with mimic-miR control (Ctr) or mimic-miR-30a **B.** or from SKOV3, HEY and OVCA433 EOC cell lines **C.** are analyzed by western blotting for ET_A_R expression. β-actin is used as loading control. **D.** Expression levels of ET_A_R and miR-30a in a panel of seven EOC cell lines. The ratio of ET_A_R/β-actin, evaluated by western blotting as shown in B and C, and miR-30a/U6 expression, evaluated by qPCR, is shown as bar and line, respectively. **E.** Scatter plot of the expression of miR-30a, as determined by qPCR, in the 39 EOC patients dichotomized into ET_A_R high or ET_A_R low expressing tumors. The expression levels of miR-30a were normalized to RNU44 expression. Horizontal lines indicate median *n* = 13 (low ET_A_R), *n* = 26 (high ET_A_R) and Mann-Whitney ran sum test was used in the statistical analysis (*p* = 0.0081).

In order to identify miRNA able to bind the ET_A_R 3′UTR, we performed a computational target prediction analysis by using TargetScan [28], miRanda [29] and miRDB [30] bioinformatic algorithms (Supplementary Figure S1A). ET_A_R 3′UTR contained a perfect complementary matching region at nucleotides 1327-1334, 2200-2205, 2321-2327 for miR-30 family (Supplementary Figure S1B), consisting of six distinct mature miRNA sequences: miR-30a/miR-30c-2, miR-30d/miR-30b, and miR-30e/miR-30c-1. To search for the critical miR-30 family members involved in aberrant ET_A_R expression reported in chemoresistant EOC cells [4, 8], we firstly analyzed their expression levels in 2008 and A2780 human cell lines and in their cisplatinum-resistant variants. Real-time PCR (qPCR) experiments demonstrated that miR-30a resulted significantly downregulated in both 2008 CIS and A2780 CIS cells (Figure 1A and Supplementary Figure S1C), expressing higher mRNA and protein levels of ET_A_R compared to sensitive cells (Figure 1B and Supplementary Figure S1D). It is notable that miR-30a expression was also downregulated in paclitaxel-resistant A2780 cell line, which overexpressed ET_A_R [4,8] (Supplementary Figure S1D and S1E), demonstrating that overexpression of miR-30a directly binds ET_A_R, which is responsible for regulating the rate limiting of both paclitaxel and cisplatinum response.

In a panel of EOC cell lines, the expression of miR-30a was substantially decreased and was associated with a highy expression of ET_A_R (*p* = 0.02) (Figure 1B, 1C and 1D). The ectopic introduction of miR-30a significantly reduced ET_A_R protein levels, even in resistant cells expressing low miR-30a and high ET_A_R (Figure 1B). miR-30a did not affect the expression of ET_A_R mRNA (Supplementary Figure S1F), indicating that it unlikely inhibits transcription. Furthermore, we evaluated the expression of miR-30a by qPCR in a cohort of 39 EOC human tumor samples, whose clinical-pathological characteristics are summarized in Supplementary Table S1, and in which the ET_A_R expression has been previously examinated by immunohistochemistry (IHC) [8]. As show in Figure 1E, we found a significant (*p* = 0.0081) inverse correlation between the expression levels of ET_A_R and miR-30a. The median expression value of miR-30a, normalized for RNU44 expression, was significantly lower in tumors expressing high (*n* = 26) versus low (*n* = 13) ET_A_R levels (median=8.4, range 0.3-21,203 vs median=54 range 3.3-4,045, respectively). These data suggest that the regulation of miR-3oa/ETAR axis is involved in the pathobiology of EOC.

### ET_A_R is a novel target of miR-30a

**Figure 2:**
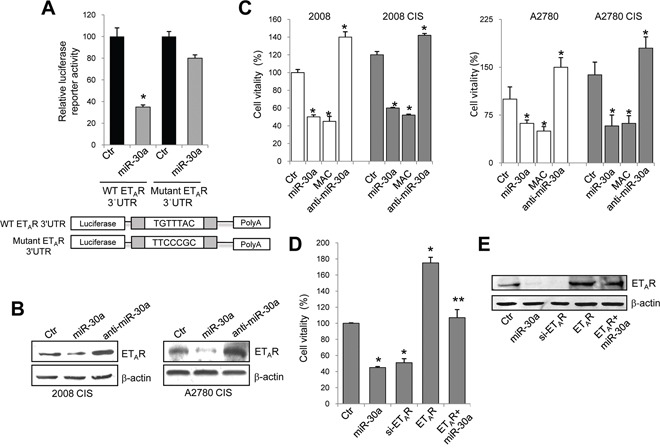
ET_A_R is a novel target of miR-30a **A.** Luciferase activity in 2008 cells cotransfected with mimic-miR control (Ctr) or mimic-miR-30a and the reporter plasmid containing 3′UTR region of ET_A_R (WT ET_A_R 3′UTR) and its mutant (mutant ET_A_R 3′UTR). Values are the mean ± SD (*n* = 3; *, *p* < 0.05 compared to Ctr). **B.** Western blotting for ET_A_R in the lysates from 2008 CIS and A2780 CIS cells transfected for 48h with mimic-miR control, mimic-miR-30a or anti-miR-30a. β-actin is used as loading control. **C.** Cell viability of sensitive and resistant 2008 and A2780 cells, transfected as in B or treated with macitentan (MAC). Values are the mean ± SD (*n* = 3; *, *p* < 0.01 vs Ctr). **D.** Cell vitality of 2008 CIS cells, transfected with control plasmid alone (Ctr), or with mimic-miR-30a, or si-ET_A_R, or with ET_A_R expression plasmid alone or in combination with mimic-miR30a. Values are the mean ± SD (*n* = 3; *, *p* < 0.05 compared to Ctr; **, *p* < 0.05 compared to miR-30a). **E.** Lysates of cells treated as in D are analyzed by western blotting for ET_A_R expression. β-actin is used as loading control.

To assess whether ET_A_R is a direct target of miR-30a, we utilized a luciferase report assay. miR-30a significantly reduced ET_A_R 3′UTR reporter activity, confirming that miR-30a directly binds the ET_A_R mRNA (Figure 2A). miR-30a did not affect luciferase activity with ET_A_R 3′UTR possessing a mutation in the conserved miR-30a binding site (1327-1334) (Figure 2A). Taken together, these results suggest that miR-30a directly decreases ET_A_R expression through sequence-specific binding with 3′UTR of ET_A_R mRNA. In order to further confirm miR-30a specificity in ET_A_R regulation, we transfected chemoresistant EOC cells with the antago-miR-30a (anti-miR-30a), chemically engineered oligonucleotides used to silence endogenous miR-30a. Importantly, increased levels of ET_A_R protein was observed in miR-30a-silenced cells compared to control cells (Figure 2B). Next, we used miR-30a overexpression and inhibition strategies in cell proliferation. The ectopic introduction of miR-30a significantly decreased cell vitality of both sensitive and resistant EOC cells. To determine whether ET_A_R inhibition can recapitulate the effects of miR-30a observed in EOC cells, we explored molecular ET_A_R targeting treatment by using the small molecule macitentan, a potent ET_A_R antagonist with significant affinity for ET_B_R. Following treatment with macitentan, the cell vitality was significantly decreased. On the contrary, the silencing of miR-30a enhanced the proliferation of these cells (Figure 2C). Silencing of ET_A_R mimicked the effect of miR-30a inhibiting cell vitality (Figure 2D and 2E). Importantly, ectopic introduction of miR-30a in EOC cells overexpressing ET_A_R, with an expression vector encoding ET_A_R lacking 3′UTR, was unable to completely suppress cell proliferation (Figure 2D and 2E). Altogether, these findings demonstrate that miR-30a functionally binds the ET_A_R 3′UTR, thereby inhibiting ET_A_R expression and cell proliferation.

### Overexpression of miR-30a sensitizes EOC cells to cisplatinum-induced apoptosis

**Figure 3:**
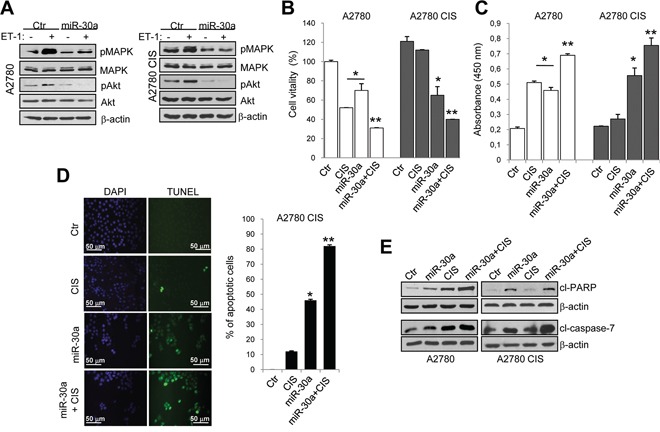
Ectopic expression of miR-30a sensitizes EOC cells to cisplatinum-induced apoptosis **A.** Lysates from A2780 and A2780 CIS cells transfected with Ctr or mimic-miR-30a and stimulated with ET-1 as indicated, are immunoblotted with anti-pMAPK, anti-MAPK, anti-pAkt and anti-Akt Abs. β-actin is used as loading control. **B.** Cell vitality of sensitive and resistant A2780 cells transfected with Ctr or mimic-miR-30a and treated with cisplatinum (CIS; 1 μM) for 72 h alone or in combination. Values are the mean ± SD (*n* = 3; *, *p* < 0.05 vs Ctr; **, *p* < 0.05 vs cisplatinum-treated cells). **C.** Detection of DNA fragmentation in sensitive and resistant A2780 cells cultured for 72 h as indicated in B. Values are the mean ± SD (*n* = 3; *, *p* < 0.05 vs Ctr; **, *p* < 0.05 vs cisplatinum-treated cells). **D.** TUNEL immunofluorescence staining for apoptotic cells (green) treated for 72 h as indicated in B. Cell nuclei are counterstained with DAPI (blue). Graph represents the percentage of apoptotic cell numbers. Magnification x40. Values are the mean ± SD (*n* = 3; *, *p* < 0.05 vs Ctr; **, *p* < 0.05 vs cisplatinum-treated cells). **E.** Western blotting for cleaved PARP (cl-PARP) and caspase-7 (cl-caspase-7) in sensitive and resistant A2780 cells transfected and treated as indicated in B. β-actin is used as loading control.

We next evaluated whether miR-30a could inhibit the survival pathways activated by ET-1/ET_A_R axis to protect tumor cells from drug-induced apoptosis [4]. miR-30a-mediated ET_A_R inhibition was accompanied with reduced ET-1-induced phosphorylation of both p42/p44 MAPK and Akt in A2780 sensitive and cisplatinum-resistant cells (Figure 3A). The analysis of cell viability (Figure 3B and Supplementary Figure S2A) and DNA fragmentation (Figure 3C and Supplementary Figure S2B) showed that the addition of cisplatinum in cells overexpressing miR-30a, lead to an enhanced sensitivity to the chemotherapeutic drug not only in sensitive but also in resistant cells. In line with these data, by performing a TUNEL assay in A2780 CIS cells, we observed a significant increase in the number of apoptotic cells following the overexpression of miR-30a (46%) (Figure 3D). Moreover, the addition of cisplatinum in cells transfected with miR-30a sensitized chemoresistant cells to cisplatinum-induced apoptosis (82%). As shown in Figure 3E, overexpression of miR-30a in A2780 sensitive and resistant cells enhanced the cleavage of poly (ADP-ribose) polymerase (PARP) and caspase-7, compared to the control cells. Importantly, the activation of these apoptotic markers was more pronounced in the presence of cisplatinum. Together these results demonstrate that overexpression of miR-30a re-sensitizes resistant EOC cells to cisplatinum by targeting ET_A_R.

### Overexpression of miR-30a reverts EMT phenotype and impairs cell invasion and plasticity, and VEGF production

**Figure 4:**
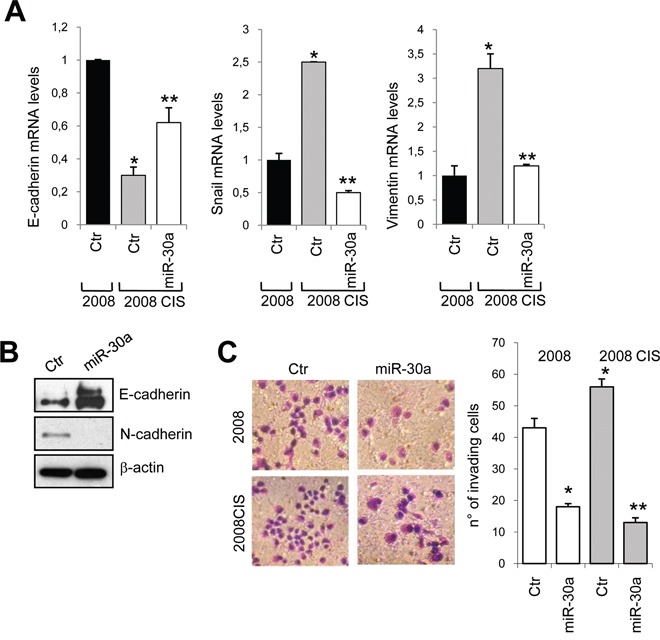
miR-30a inhibits EMT phenotype and cell invasion **A.** E-cadherin, Snail and vimentin mRNA expression in 2008 or 2008 CIS cells transfected with Ctr or mimic-miR-30a evaluated by qPCR. Cyclophilin-A is used to normalize. Values are the mean ± SD (*n* = 3; *, *p* < 0.05 vs Ctr of sensitive cells; **, *p* < 0.05 vs Ctr of resistant cells). **B.** Lysates from resistant 2008 CIS cells transfected with Ctr or mimic-miR-30a are analyzed by western blotting for E-cadherin and N-cadherin expression. β-actin is used as loading control. **C.** Chemoinvasion assay of 2008 and 2008 CIS cells transfected with Ctr or mimic-miR-30a. The crystal violet-stained invasive cells are photographed (*left*) or counted (*right*). Magnification x40. Columns show the mean ± SD (*n* = 3; *, *p* < 0.001 vs Ctr of sensitive cells; **, *p* < 0.001 vs Ctr of resistant cells).

**Figure 5:**
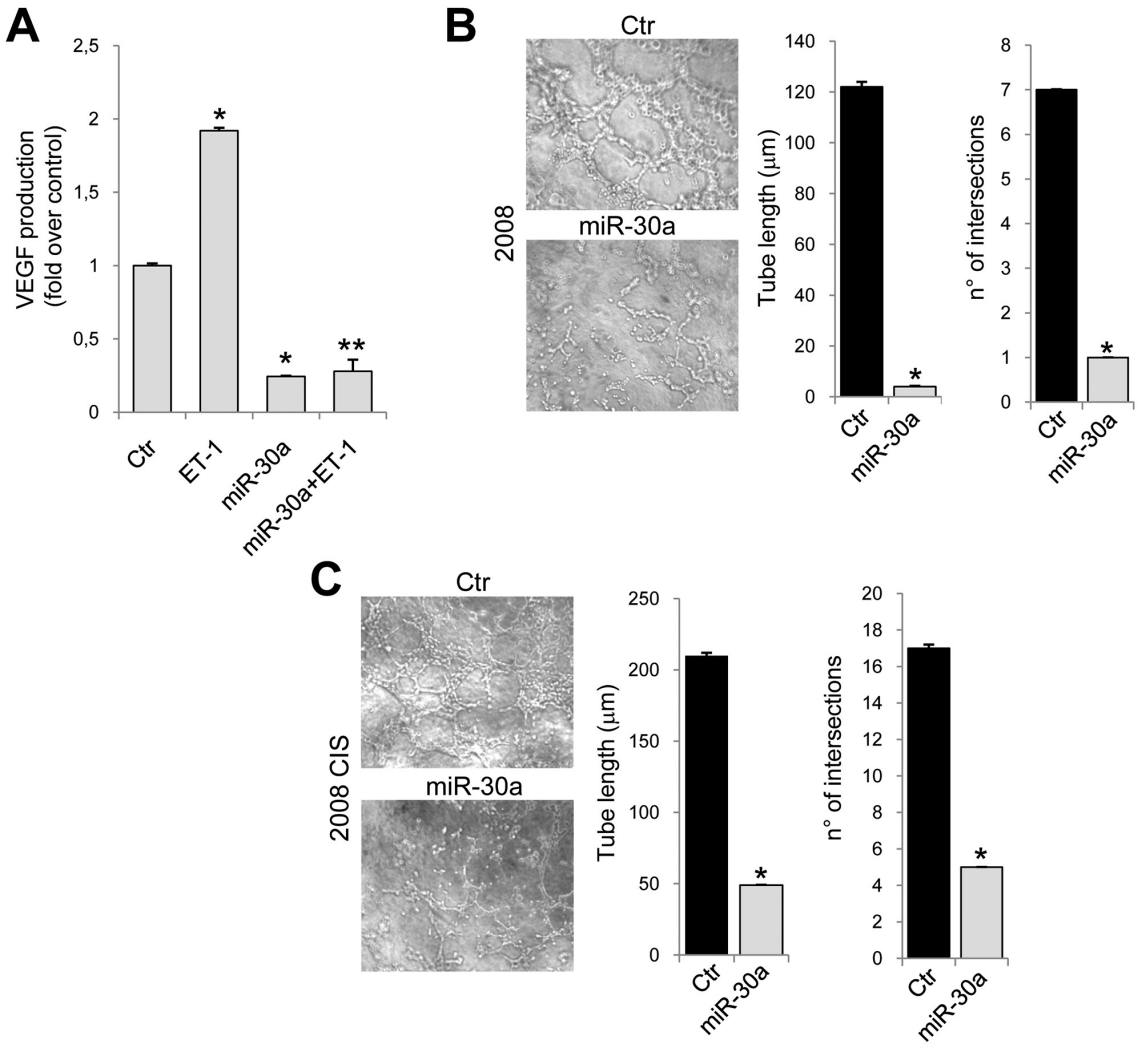
miR-30a inhibits the release of VEGF and vasculogenic-like tubule formation **A.** VEGF release evaluated by ELISA from conditioned media of 2008 CIS cells transfected with Ctr or mimic-miR-30a in the presence or in the absence of ET-1. Values are the mean ± SD (*n* = 3; *, *p* < 0.001 vs Ctr; **, *p* < 0.001 vs ET-1-treated cells). Tubule-like structure formation in 2008 **B.** or 2008 CIS **C.** cells transfected with Ctr or mimic-miR-30a was photographed (*left*) and counted (*right*). Magnification x20. Graphs represent the tube length and the number of intersections. Values are the mean ± SD (*n* = 3; *, *p* < 0.05 vs Ctr).

ET-1/ET_A_R overexpression, by regulating EMT and invasive behavior, endows EOC cells with an increased survival capacity and resistance to chemotherapeutic agents [4]. 2008 and A2780 resistant cells expressed enhanced mRNA levels of Snail and vimentin, associated with a concomitant decrease of E-cadherin expression (Figure 4A and Supplementary Figure S3). The overexpression of miR-30a restored E-cadherin levels, and inhibited the expression of both vimentin and Snail (Figure 4A and Supplementary Figure S3). As determined by western blotting analysis, in resistant 2008 cells miR-30a overexpression enhanced E-cadherin and reduced N-cadherin protein expression (Figure 4B), indicating that this miRNA is able to revert the EMT phenotype in EOC cells. In line with above results, transfection with miR-30a resulted in a strong inhibition of cell invasion of sensitive as well as chemoresistant 2008 cells (Figure 4C). Because in EOC cells, ET-1 through ET_A_R induces the release of vascular endothelial growth factor (VEGF) [31], which has been reported to have important regulatory role in drug-sensitivity [22], we evaluated whether miR-30a was also able to modulate the release of VEGF by EOC cells. Importantly, miR-30a transfection in 2008 CIS cells reduced the basal, as well as the ET-1-induced VEGF production (Figure 5A), demonstrating that regulation of the miR-30a/ET_A_R axis is also important in the release of the angiogenic factor VEGF. Aggressive EOC cells, expressing high levels of EMT associated markers, are capable to form vasculogenic mimicry [32]. Consistently, we found that 2008 and 2008 CIS cells plated on matrigel formed a network of interconnected tubules (Figure 5B and 5C). Notably, quantification analysis demonstrated that the length of tubes and the number of their intersections in chemoresistant EOC cells increased significantly compared to sensitive cells. Cells overexpressing miR-30a were unable to form elongated structures and formed fewer intersections (Figure 5B and 5C), indicating that ET-1/ET_A_R pathway is implicated in the *in vitro* formation of vasculogenic-like networks in chemoresistant EOC cells, and that miR-30a inhibits EMT, cell invasion and plasticity.

### Tumor-inhibitory effects of miR-30a in resistant EOC xenografts

**Figure 6:**
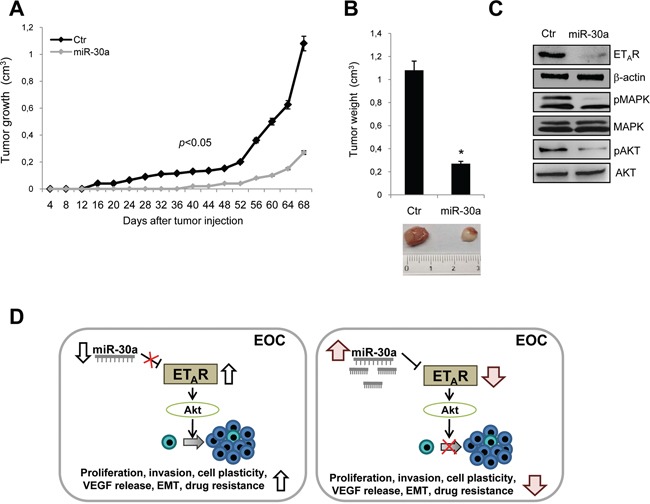
Tumor-inhibitory effects of miR-30a in resistant EOC xenografts **A.** Growth curve of tumors from vector control (Ctr) or miR-30a-expressing resistant 2008 CIS cells s.c. injected into the mice. Data points, averages ± SD (*p*<0.05). **B.** Tumor weight of tumors grown in vector control (Ctr) and miR-30a 2008 CIS xenografts reported as the mean ± SD. *, *p* < 0.05 vs Ctr. *Bottom*, representative tumors of 2008 CIS xenografts. **C.** Western blotting analysis of ET_A_R, p-MAPK, MAPK, p-Akt and Akt expression in tumors of vector control (Ctr) and miR-30a 2008 CIS xenografts. β-actin is used as loading control. **D.** A schematic model describing the potential mechanism underlying the suppressive role of miR-30a in EOC. In chemoresistant EOC cells downregulated miR-30a expression has strong increased effects on ET_A_R expression promoting enhancement of Akt and MAPK signaling activation. Therefore miR-30a downregulation results in increased cell proliferation, invasion, plasticity, VEGF release, EMT and drug resistance by inhibiting ET_A_R pathway.

Next we explored the effect of miR-30a on tumorigenesis *in vivo*, by using the 2008 CIS cells stably transfected with miR-30a or with vector control (Ctr) (Supplementary Figure S4). After cells were injected subcutaneously into the flank of nude mice, tumor volume was monitored every 4 days and the tumor growth curves were plotted. Tumor weight from the miR-30a overexpressing group was significantly reduced (70%) when compared with control group (*p* < 0.05; Figure 6A and 6B). Moreover, the control group exhibited faster growth rate. In parallel, western blotting analysis showed lower levels of ET_A_R and a significant reduction of MAPK and Akt activation in miR-30a overexpressing mice compared with control (Figure 6C). These findings demonstrate that overexpression of miR-30a results in decreased tumor growth in chemoresistant EOC xenografts.

### miR-30a correlates with poor survival in TCGA of ovarian cancer

**Figure 7:**
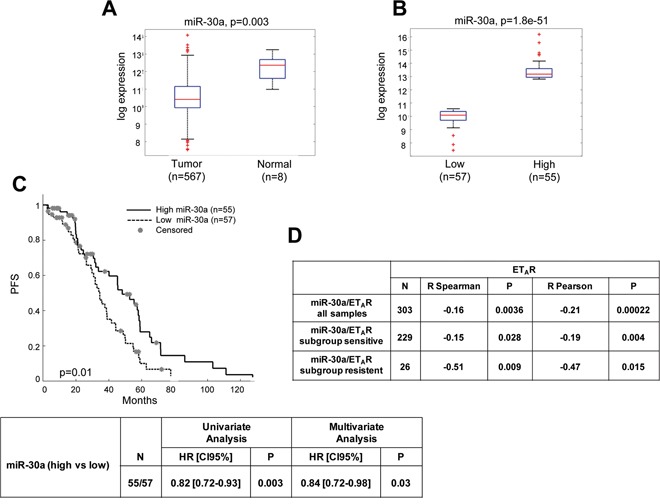
miR-30a correlates with poor survival in TCGA of ovarian cancer **A.** Boxplot distribution of miR-30a expression in 567 tumors and 8 normal samples. **B.** Boxplot distribution of miR-30a expression in 112 patients subdivided into low or high miR-30a by using z-score higher than 1 (*n* = 55) and lower than −1 (*n* = 57). **C.** Kaplan-Meier curves of progression free survival for subgroup of 112 patients described in B. Univariate and multivariate analysis by Cox proportional hazard regression for subgroup of patients described in B, adjusted for stage, grade, and treatment response. **D.** ET_A_R gene expression resulted to be inversely correlated to miR-30a expression in the TCGA cohort of HG-SOC patients (*n* = 303 tumoral samples). A significative inverse correlation was found in both sensitive and resistant samples.

To assess the clinical relevance of miR-30a, we analyzed its expression in TCGA of HG-SOC patient cohort. We found a strong downregulation of miR-30a when comparing tumoral (*n* = 567) and non tumoral (*n* = 8) samples in this cohort (*p* = 0.003; Figure 7A). To evaluate whether mir30a downregulation has a prognostic value, we subdivided patients of TCGA cohort into low or high miR-30a by using z-score higher than 1 (*n* = 112). As shown in Figure 7B, low miR-30a expression was documented in 57 HG-SOC patients (51%) and high miR-30a expression in 55 HG-SOC patients (49%; *p* = 1.8e-51). Of clinical relevance, the TCGA data set supported the association between low miR-30a expression and poor patient outcome (*p* = 0.01) (Figure 7C). Univariate and multivariate analysis revealed the prognostic role of low miR-30a expression that was independent from other clinical variables (tumor stage, grade and treatment response) (Figure 7C). Interestingly, ET_A_R gene expression inversely correlated with miR-30a expression in TCGA dataset. This anticorrelation was also present when comparing sensitive (*n* = 229) and resistant (*n* = 26) sample patients (Figure 7D). This might suggest that the overexpression of ET_A_R due to the loss of miR-30a activity might contribute to chemoresistance onset. In line with these observations, we observed a negative correlation between miR-30a and ET_A_R, evaluated by IHC in our sample set of 39 EOC (*p* = 0.039; Supplementary Table S2), in which patients were dichotomized into low or high miR-30a expression groups by using the median value as the cut-off. Next, we analyzed whether expression of miR-30a could correlate with survival analysis in our sample set. Recurrence/progression of disease was observed in 30 cases (76.9%), while death of disease was documented in 26 patients (66.7%); Supplementary Figure S5 shows the progression-free survival (PFS) curves according to miR-30a expression; the 3-year PFS was 38% in patients exhibiting high miR-30a expression versus 10% in cases with low miR-30a expression (*p* = 0.043).

## DISCUSSION

An in depth understanding of the molecular drivers of EOC chemoresistance is of paramount importance for improving the therapeutic regimens. We recently reported that high levels of ET_A_R expression are associated with poor prognosis and chemoresistance in EOC [8], however, the molecular mechanisms mediating chemoresistance remain poorly understood. In this study we provide the first evidence that downregulation of miR-30a is a feature of chemoresistant EOC cells. This decrease is likely to contribute to the chemoresistance by inhibiting chemotherapy-induced apoptosis through upregulation of ET_A_R and sequential activation of the MAPK and Akt pathways. Transfection of miR-30a sensitized chemoresistant cells to cisplatinum by promoting apoptosis. Importantly, we provide preclinical experiments setting the scene for a novel miR-30a function as a tumor suppressor in chemoresistant EOC by targeting ET_A_R signaling axis. Abundant ET_A_R expression and low expressed miR-30a enhance the acquisition of an aggressive chemoresistant potential of EOC cells. The investigation of the mechanism of miR-30a in EOC reveals that ET_A_R can be post-transcriptionally regulated by miR-30a. Several studies have demonstrated that ET_A_R signaling contributes to the acquisition of chemoresistance and EMT [3–5, 8, 33, 34]. In addition, ET-1 is present at high levels in tumor ascites [35] and high grade ovarian tumors overexpress ET_A_R [27, 36], thus making it imperative to understand the effect of ET-1/ET_A_R axis on ovarian tumor biology and the factors that regulate this signaling pathway. A comprehensive analysis recently highlighted that ET_A_R overexpression is associated with worse survival in the groups of proliferative and mesenchymal HG-SOC [27]. In this context, we recently reported that ET_A_R co-opts Wnt/Δ-catenin signaling, which is a critical driver of EOC chemoresistance [37], to acquire a chemoresistant and mesenchymal phenotype, thus sustaining tumor progression through a self-strengthening feedback loop [8]. Here we have shown that miR-30a inhibits EOC cell invasion, plasticity, and the release of the pro-angiogenic factor VEGF, that characterize the chemoresistant behavior and EMT (Figure 6D).

Several *in vitro* and *in vivo* studies have shown that miRNA can modulate the sensitivity of EOC to chemotherapy [21–24]. The most frequently de-regulated miRNA in chemoresistant EOC are involved in EMT, as let-7 and miR-200 families [20], or in the receptor signaling pathways, as miRNA-31 that targets MET [23]. High-throughput analysis of the miRNA profile in a panel of drug-resistant EOC cells revealed that miR-30c, miR-130a and miR-335 were downregulated [21]. Moreover, a miRNA signature defines EOC chemoresistance through modulation of angiogenesis, including miR-484 as regulator of VEGF pathway [22].

miR-30 family members are located on human chromosome 6q.13 and are among the most dysregulated miRNA in metastatic cancer [38, 39]. Although recent findings suggest that miR-30 family functions as tumor suppressor in many human cancer, including gastric, anaplastic thyroid, breast, and non-small-cell lung cancers [25, 26, 40–42], its association with drug resistance has not been elucidated. Our study highlights a hitherto unappreciated role of miR-30a in EOC drug resistance and EMT and defines a unique mechanism by which it directly targets ET_A_R signaling axis and contributes to poor patient outcome. In parallel, inhibition of ET_A_R activity, by the approved small molecule macitentan recapitulated the effect of miR-30a overexpression, indicating that ET_A_R target therapy should represent a novel strategy to overcome chemotherapy resistance. Consistent with *in vitro* results, we observed that expression of miR-30a delayed tumor formation in xenograft tumors, suggesting that miR-30a is one of the tumor suppressor miRNA and that miR-30a-ET_A_R axis plays a crucial role in EOC cell survival. Accordingly, this miRNA was downregulated in tumor samples of the TCGA HG-SOC cohort relative to non-tumoral ovarian tissues. Of translational relevance, in this TCGA dataset low miR-30a expression correlates with poor outcome. Multivariante analysis showed that miR-30a expression had predictive values for overall survival identifying low expression of miR-30a as an independent prognostic factor. In line with these results, analysis performed in our set of EOC tissues suggest that low miR-30a is associated with poor prognosis. Moreover, the inverse correlation between miR-30a and ET_A_R expression further highlights the pathobiologic relevance of miR-30/ET_A_R axis in EOC. These findings are highly supported by our previous analysis performed on the same cohort of EOC patients demonstrating that the association between ET_A_R expression and poor survival is to be ascribed to the unfavorable prognostic role of ET_A_R in the subset of platinum-resistant cases [8]. In conclusion, our results reveal a novel mechanism in which miR-30a suppresses EOC aggressiveness and chemoresistance by inhibiting ET_A_R expression at the post-transcriptional level, and widening the horizon about the regulatory mechanism of ET_A_R. These findings also suggest that miR-30a/ET_A_R axis may serve as possible target to improve EOC therapeutic outcome. In light of our findings, the use of miR-30a to predict prognosis and clinical response to chemotherapy warrants future evaluation in a large cohort of patients with EOC.

## MATERIALS AND METHODS

### Cells and cell culture conditions

The human ovarian carcinoma cell line A2780 was obtained from European Collection of Cell Cultures (Salisbury, UK). To retain cisplatinum (CIS) and taxol (TAX) resistance, 1 μM cisplatinum (Pfizer, Italy) and 50 nM paclitaxel (Bristol Myers, Italy) were added to the culture medium A2780 CIS and A2780 TAX, respectively, every two passages. The 2008 cell line and its cisplatinum-resistant subclone 2008C13 (CIS) were kindly provided by Dr. S.B. Howell (University of San Diego, La Jolla, CA, USA). Human ovarian carcinoma cell lines, HEY and OVCA433 were kindly provided by Prof. G. Scambia (Catholic University School of Medicine, Rome, Italy). The SKOV-3 cell line was obtained from the American Type Culture Collection (VA, USA). All cells, cultured as previously described [4], were passed in our laboratory for fewer than 3 months after resuscitation and were tested routinely for cell proliferation as well as mycoplasma contamination, and they showed similar growth rate and negative mycoplasma during the experiments. Cells were serum starved by incubation in serum-free medium for 24 h. ET-1 was used at 100 nM and was purchased from Bachem (Switzerland). Macitentan, also called ACT-064992 or N-(5-[4-bromophenyl]-6-{2-[5-bromopyrimidin-2-yloxy]ethoxy}pyrimidin-4-yl)-N′-propylsulfamide, was added 30 min before ET-1 at a dose of 1 μM and was kindly provided by Actelion Pharmaceuticals, Ltd. (Switzerland).

### RNA isolation and quantitative real-time polymerase chain reaction (qPCR)

Total RNA was extracted using the Trizol reagent (Life Technologies, Italy) according to the manufacturer's protocol. First-strand complementary DNA was synthesized using SuperScript^®^ VILO™ cDNA synthesis kit (Life Technologies). qPCR was performed using Power SYBR Green PCR Master Mix (Applied Biosystems) with a 7500 Fast Real-Time PCR System (Applied Biosystems) according to the manufacturer's instructions. The expression levels of ET_A_R, E-cadherin, Snail, and vimentin were determined by normalizing to cyclophilin-A mRNA expression. The primers employed for qPCR were as follows: ET_A_R Fw: 5′-GGGATCACCGTCCTCAACCT-3′; ET_A_R Rev: 5′-CAGGAATGGCCAGGATAAAGG-3′; E-cadherin Fw: 5′-CCCACCACGTACAAGGGTC-3′; E-cadherin Rev: 5′-ATGCCATCGTTGTTCACTGGA-3′; Snail Fw: 5′-CTTCCAGCAGCC-CTACGAC-3′; Snail Rev: 5′-CGGTGGGGTTGAGGATCT-3′; vimentin Fw: 5′-TTTGAAG-AAACTCCACGAAGAGGA-3′; vimentin Rev: 5′-CCACATCGATTTGGACATGCT-3′; cyclophilin-A Fw: 5′-TTCATCTGCACTGCCAAGAC-3′; cyclophilin-A Rev: 5′-TCGAGTT-GTCCACAGT CAGC-3′. Isolation of total RNA or miRNA fraction from human cryostatic EOC sections was performed by using the Isolation of small and large RNA kit (Macherey-Nagel, Germany). For miRNA analysis RNA was reverse transcribed using the hsa-miR-30a, hsa-miR-30b, hsa-miR-30c, hsa-miR-30d, hsa-miR-30e, U6 snRNA and RNU44 TaqMan™ MicroRNA Assay systems (Applied Biosystems, NJ, USA) and pPCR was performed by using Kapa Probe Fast qPCR Master Mix (Kapa Biosystems, MA, USA). miRNA levels were normalized to U6 snRNA or RNU44 expression. Final data were obtained by using 2^−ΔΔCt^ method [43].

### Western blot analysis

Whole cell lysates were prepared using a modified RIPA buffer (50 mM Tris-HCl pH 7.4, 250 mM NaCl, 1% Triton X-100, 1% sodium deoxycholate, 0.1% SDS) containing a mixture of protease and phosphatase inhibitors. Total proteins were subjected to SDS-PAGE, and the antibodies (Abs) used for the study were as follows: anti-ET_A_R and anti-β-actin (C-11), purchased from Abcam (UK) and Santa Cruz Biotechnology (CA, USA), respectively; anti-p-p44/42 MAPK (Thr202/Tyr204), anti-p44/42 MAPK, anti-pAkt (S473), anti-Akt, anti-cleaved-PARP (Asp214), and anti-cleaved-caspase 7 (Asp198), were from Cell Signaling Technology (MA, USA). Anti-E-cadherin and anti-N-cadherin were purchased from BD Biosciences (CA, USA). Western blotting filters were developed using the ECL-plus detection system or, otherwise, the SuperSignal West Femto kit (Thermo Scientific, IL, USA). Western blots for ET_A_R were quantified by densitometric analysis using ImageJ software.

### Transient and stable transfection

Cells were transiently transfected with Syn-hsa-miR-30a-5p (mimic-miR-30a), anti-hsa-miR-30a-5p (anti-miR-30a), hs-EDNRA FlexiTube siRNA (pool of 4 siRNAs; si-ET_A_R) or AllStars Negative Control siRNA (Qiagen, Germany) using RNAi/MAX Lipofectamine (Life Technologies) at a final concentration of 50 nM according to the manufacturer's suggestions. Otherwise, cells were transiently transfected with 1 μM of EDNRA plasmid (Origene, MD, USA) or pGL3-Basic Control plasmid (Promega, WI, USA) using Lipofectamine 2000 (Life Technologies) according to the manufacturer's instructions. Stable clones were generated by transfecting cells with pCMV-miR vector control (Ctr) or miR-30a expression vector (Origene) using Lipofectamine 2000 and cells were then selected with 700 μg/ml G418 sulfate (Calbiochem-Novabiochem Corporation, CA, USA). The exogenous miR-30a over the endogenous counterpart (vector alone) was monitored by qPCR (Supplementary Figure S3).

### Luciferase reporter gene assay

Cells cultured in 12-well plate were transiently transfected with 50 nM of mimic-miR-30a or miR-negative control together with 0.5 μg of ET_A_R 3′UTR reporter plasmid (Origene) or its mutant (ET_A_R 3′UTR 1328-1332) by using Lipofectamine 2000 (Life Technologies) according to manufacturer's instructions. Transfection efficiency was monitored by cotransfection with 0.1 μg of pmiR-Report Beta-gal (Life Technologies), which was used as an internal control. Reporter activity was measured after 48h using the Luciferase assay system (Promega) and normalized to β-galactosidase activity. ET_A_R 3′UTR reporter plasmid mutagenesis was performed using the QuikChange Site-Directed Mutagenesis Kit (Stratagene, CA, USA) and the following primers: Fw: 5′-ACAGTGACTTTTGCTGGGCA TTTTCCCAGATTCCCGCAGACTGTGAGTACAGCA G-3′; Rev: 5′-CTGCTGTACTCACAGTCTGCGGGAATCTGGGAAAATGCCCAGCAAAAGTCACT-GT-3′.

### Cell viability analysis

Sensitive or cisplatinum resistant cells were seeded in twelve-well plates in triplicate. After 24 h cells were transfected with miRNA mimics (miR-negative control, mimic-miR-30a, anti-mimic-miR-30a, si-ET_A_R or ET_A_R plasmid) and/or treated with cisplatinum and macitentan. After 48-72 h, cells were harvested and counted by using trypan blue dye exclusion method.

### Apoptosis assays

Terminal deoxynucleotidyl transferase dUTP nick end labeling (TUNEL) assay was performed using a commercial apoptosis detection kit (Roche, Italy) according to the manufacturer's suggestions. Breafly, treated cells were washed in PBS, fixed in 4% Paraformaldehyde (PFA), and permabilized with 0.1% sodium citrate, 0.1% Triton X-100 in 1xPBS for 2 minutes at 4°C. Nuclei were stained with DAPI (Vector Laboratories, CA, USA) and slides were mounted with Vectashield mounting medium for fluorescence (Vector laboratories). Fluorescence signals were captured at 40X magnitude by using a Leica DMIRE2 microscope equipped with a Leica DFC 350FX camera and elaborated by a Leica FW4000 deconvolution software (Leica, Germany). The percentage of apoptotic cells was calculated by the ratio of TUNEL-positive nuclei and total nuclei stained with DAPI. Quantitative detection of apoptotic cells was also performed on 1×10^6^ cells using TiterTACS colorimetric apoptosis detection kit (Trevigen, MD, USA) according to the manufacturer's instructions.

### Chemoinvasion assay

Chemoinvasion assays were carried out using modified Boyden chamber consisting of transwell membrane filter inserts with 8 μm size polycarbonate membrane precoated with polymerized collagen placed in a 24-well plate (BD Biosciences). The cells (5 × 10^5^ cells) were stimulated with serum-free RPMI, or RPMI containing ET-1, added to the lower chamber. The cells were left to migrate for 12 h at 37°C. Cells on the upper part of the membrane were scraped and the migrated cells were fixed in 3.7% PFA and stained with 0.4% Crystal Violet in 10% ethanol. From every transwell, several images were taken under a phase-contrast with Olympus Ix70 microscope (Olympus Corporation, Japan) at 10x magnification and two broad fields were considered for quantification.

### Tubule-like structure formation

The ability of tumor cells to form capillary-like structure formation has been assessed on cells cultured on Cultrex (basal membrane extract; Trevigen, MD, USA), as previously described [32]. Images were analyzed with ImageJ v.1.34s (http://rsb.info.nih.gov/ij/) for determining the length of the tubes and the number of intersections. Representative images were captured with Olympus Ix70 microscope at 20x magnification.

### ELISA

Cells were plated at 4 × 10^5^ per 60 mm dish and transfected for 48h with mimic-miR control or mimic-miR-30a in serum-free medium. Then, cells were treated or not with ET-1 for 24h. The VEGF protein levels in the cell-conditioned medium were determined in triplicate by ELISA using the Quantikine human VEGF immunoassay kit (R&D Systems, Minneapolis, MN, USA). The sensitivity of the assay is less than 5.0 pg/ml.

### Xenografts in nude mice

2 × 10^6^ viable 2008 cisplatinum-resistant control vector or miR-30a stable expressing cells were randomly injected subcutaneously into the flank of female athymic (nu+/nu+) of 5 mice for group, 4–6 week of age (Charles River Laboratories, Italy), following the guidelines for animal experimentation of the Italian Ministry of Health. Tumor volume was measured with caliper and was monitored every 4 days and the tumor growth curves were plotted. Tumor volume was calculated using the formula: π/6 larger diameter × (smaller diameter)^2^. At the end of experiments all the mice were euthanized, the site of tumors was noted, and the removed tumors were frozen and analyzed for western blotting.

### Samples from human EOC tissues

Human tumor and normal ovarian specimens were kindly provided by Prof. G. Ferrandina from patients admitted to the Gynecologic Oncology Unit, Catholic University of Campobasso and Rome (Italy). Human EOC specimens were collected at time of initial diagnosis both in patients undergoing maximal cytoreduction and in patients deemed unresectable and therefore triaged to neo-adjuvant chemotherapy; therefore all tissues specimens were naive to chemotherapy. Tissues specimens were immediately frozen in liquid nitrogen and then stored at −80°C until the assay. Written informed consent to tumor tissue collection was obtained by each patient according to the research protocol approved by the local ethical committee.

### Immunohistochemistry

Immunohistochemical analysis of ovarian cancers was performed on archival from 39 frozen tumors collected from patient population above described. Avidin-biotin indirect immunoperoxidase staining was performed by using the polyclonal antibody anti-ET_A_R (Abbott Laboratories, IL, USA). The avidin-biotin assays were performed using the Vectastain Elite kit (Vector Laboratories). Sections in which the incubation with the primary Ab was substituted by isotype-matched immunoglobulins were used as control. AEC was used as chromogenic substrate and Mayer's haematoxylin as nuclear counterstain [8].

### Bioinformatic analysis

Normalized miRNA and gene expression were obtained from TCGA of HG-SOC. Data from different platforms were interrogated (Agilent, RNAseqv2 and Illumina miRNA-Seq). All the expression value differences between subgroups of samples were evaluated applying unpaired t-test and permutation test. A false discovery rate procedure [44] was also included for multiple comparisons. Pearson's correlation coefficient and Spearman coefficient were calculated between miRNA and gene target expression. Survival and progression free survival were evaluated by Kaplan-Meier method [45] and a log-rank test used to establish the statistical significance of the distance between curves [46]. The impact of clinical variables on the survival curves was investigated by a multivariate Cox proportional hazard regression model [47]. Subgroup of samples in survival analyses were individuated basing on the z-scores of the signal, and considering as the most separate subgroups of samples those with absolute z-score higher than 1.

### Statistical analysis

Student's t-test (unpaired, two-tailed) was used for comparing statistical differences. Differences were considered statistically significant when *p*<0.05. The time course of tumor growth was compared across the groups using two-way ANOVA, with group and time as variables. The χ^2^ test or Fisher's exact test for proportion were used to analyze the distribution of clinical-pathological variables according to different subgroups. PFS was calculated from the date of surgery to the date of relapse or the date of the last follow-up. Medians and life tables were computed using the product limit estimate by Kaplan–Meier method. Statistical analysis was carried out using SPSS (SPSS II, SPSS Inc., IL, USA), SOLO (BMDP Statistical Software, CA, USA) and MATLAB (The MathWorks, MA, USA) software. The Mann-Whitney's U test was used to analyze differences in miRNA expression levels.

## SUPPLEMENTARY FIGURES AND TABLES



## References

[1] Siegel R, Naishadham D, Jemal A (2012). Cancer statistics, 2012. CA Cancer J Clin.

[2] Banerjee S, Kaye SB (2013). New strategies in the treatment of ovarian cancer: current clinical perspectives and future potential. Clin Cancer Res.

[3] Rosanò L, Spinella F, Bagnato A (2013). Endothelin 1 in cancer: biological implications and therapeutic opportunities. Nat Rev Cancer.

[4] Rosanò L, Cianfrocca R, Spinella F, Di Castro V, Nicotra MR, Lucidi A, Ferrandina G, Natali PG, Bagnato A (2011). Acquisition of chemoresistance and EMT phenotype is linked with activation of the endothelin A receptor pathway in ovarian carcinoma cells. Clin Cancer Res.

[5] Rosanò L, Spinella F, Di Castro V, Nicotra MR, Dedhar S, de Herreros AG, Natali PG, Bagnato A (2005). Endothelin-1 promotes epithelial-to-mesenchymal transition in human ovarian cancer cells. Cancer Res.

[6] Jazaeri AA, Awtrey CS, Chandramouli GV, Awtrey CS, Chandramouli GV, Chuang YE, Khan J, Sotiriou C, Aprelikova O, Yee CJ, Zorn KK, Birrer MJ, Barrett JC (2005). Gene expression profiles associated with response to chemotherapy in epithelial ovarian cancers. Clin Cancer Res.

[7] Helleman J, Smid M, Jansen MP, van der Burg ME, Berns EM (2010). Pathway analysis of gene lists associated with platinum-based chemotherapy resistance in ovarian cancer: the big picture. Gynecol Oncol.

[8] Rosanò L, Cianfrocca R, Tocci P, Spinella F, Di Castro V, Caprara V, Semprucci E, Ferrandina G, Natali PG, Bagnato A (2014). Endothelin A receptor/β-arrestin signaling to the Wnt pathway renders ovarian cancer cells resistant to chemotherapy. Cancer Res.

[9] Salani D, Taraboletti G, Rosanò L, Di Castro V, Borsotti P, Giavazzi R, Bagnato A (2000). Endothelin-1 induces an angiogenic phenotype in cultured endothelial cells and stimulates neovascularization in vivo. Am J Pathol.

[10] Spinella F, Garrafa E, Di Castro V, Rosanò L, Nicotra MR, Caruso A, Natali PG, Bagnato A (2009). Endothelin-1 stimulates lymphatic endothelial cells and lymphatic vessels to grow and invade. Cancer Res.

[11] Buckanovich RJ, Facciabene A, Kim S, Benencia F, Sasaroli D, Balint K, Katsaros D, O'Brien-Jenkins A, Gimotty PA, Coukos G (2008). Endothelin B receptor mediates the endothelial barrier to T cell homing to tumors and disables immune therapy. Nat Med.

[12] Kim SJ, Kim JS, Kim SW, Brantley E, Yun SJ, He J, Maya M, Zhang F, Wu Q, Lehembre F, Regenass U, Fidler IJ (2011). Macitentan (ACT-064992), a tissue-targeting endothelin receptor antagonist, enhances therapeutic efficacy of Paclitaxel by modulating survival pathways in orthototic models of metastatic human ovarian cancer. Neoplasia.

[13] Kim SJ, Kim JS, Kim SW, Yun SJ, He J, Brantley E, Fan D, Strickner P, Lehembre F, Regenass U, Fidler IJ (2012). Antivascular therapy for multidrug-resistant ovarian tumors by macitentan, a dual endothelin receptor antagonist. Transl Oncol.

[14] Coffman L, Mooney C, Lim J, Bai S, Silva I, Gong Y, Yang K, Buckanovich RJ (2013). Endothelin receptor-A is required for the recruitment of antitumor T cells and modulates chemotherapy induction of cancer stem cells. Cancer Biol Ther.

[15] Kim SJ, Lee HJ, Kim SW, Choi HJ, He J, Wu Q, Aldape KD, Weinberg JS, Yung WK, Conrad CA, Langley RR, Lehembre F, Regenass U (2015). Macitentan, a dual endothelin receptor antagonist, in combination with temozolomide leads to glioblastoma regression and long-term survival in mice. Clin Cancer Res.

[16] Bartel DP (2009). MicroRNAs: target recognition and regulatory functions. Cell.

[17] Hata A, Lieberman J (2015). Dysregulation of microRNA biogenesis and gene silencing in cancer. Sci Signal.

[18] Dahiya N, Morin PJ (2010). MicroRNAs in ovarian carcinomas. Endocr Relat Cancer.

[19] Iorio MV, Visone R, Di Leva G, Donati V, Petrocca F, Casalini P, Taccioli C, Volinia S, Liu CG, Alder H, Calin GA, Ménard S, Croce CM (2007). MicroRNA signatures in human ovarian cancer. Cancer Res.

[20] Llauradó M, Majem B, Altadill T, Lanau L, Castellví J, Sánchez-Iglesias JL, Cabrera S, De la Torre J, Díaz-Feijoo B, Pérez-Benavente A, Colás E, Olivan M, Doll A (2014). MicroRNAs as prognostic markers in ovarian cancer. Mol Cell Endocrinol.

[21] Sorrentino A, Liu CG, Addario A, Peschle C, Scambia G, Ferlini C (2008). Role of microRNAs in drug-resistant ovarian cancer cells. Gynecol Oncol.

[22] Vecchione A, Belletti B, Lovat F, Volinia S, Chiappetta G, Giglio S, Sonego M, Cirombella R, Onesti EC, Pellegrini P, Califano D, Pignata S, Losito S (2013). A microRNA signature defines chemoresistance in ovarian cancer through modulation of angiogenesis. Proc Natl Acad Sci USA.

[23] Mitamura T, Watari H, Wang L, Kanno H, Hassan MK, Miyazaki M, Katoh Y, Kimura T, Tanino M, Nishihara H, Tanaka S, Sakuragi N (2013). Downregulation of miRNA-31 induces taxane resistance in ovarian cancer cells through increase of receptor tyrosine kinase MET. Oncogenesis.

[24] Xiang Y, Ma N, Wang D, Zhang Y, Zhou J, Wu G, Zhao R, Huang H, Wang X, Qiao Y, Li F, Han D, Wang L (2014). MiR-152 and miR-185 co-contribute to ovarian cancer cells cisplatin sensitivity by targeting DNMT1 directly: a novel epigenetic therapy independent of decitabine. Oncogene.

[25] Liu Z, Chen L, Zhang X, Xu X, Xing H, Zhang Y, Li W, Yu H, Zeng J, Jia J (2014). RUNX3 regulates vimentin expression via miR-30a during epithelial-mesenchymal transition. in gastric cancer cells. J Cell Mol Med.

[26] Kumarswamy R, Mudduluru G, Ceppi P, Muppala S, Kozlowski M, Niklinski J, Papotti M, Allgayer H (2012). MicroRNA-30a inhibits epithelial-to-mesenchymal transition by targeting Snai1 and is downregulated in non-small cell lung cancer. Int J Cancer.

[27] Bell D, Berchuck A, Birrer M, Chien J, Cramer D, Dao F, Dhir R, Di Saia P, Gabra H, Glenn P, Godwin A, Gross J, Hartmann L (2011). Integrated genomic analyses of ovarian carcinoma. Nature.

[28] Lewis BP, Burge CB, Bartel DP (2005). Conserved seed pairing, often flanked by adenosines, indicates that thousands of human genes are microRNA targets. Cell.

[29] John B, Enright AJ, Aravin A, Tuschl T, Sander C, Marks DS (2004). Human MicroRNA targets. PLoS Biol.

[30] Wang X (2008). miRDB: a microRNA target prediction and functional annotation database with a wiki interface. RNA.

[31] Spinella F, Rosanò, Di Castro V, Natali PG, Bagnato A (2002). Endothelin-1 induces vascular endothelial growth factor by increasing hypoxia-inducible factor-1alpha in ovarian carcinoma cells. J Biol Chem.

[32] Sood AK, Seftor EA, Fletcher MS, Gardner LM, Heidger PM, Buller RE, Seftor RE, Hendrix MJ (2001). Molecular determinants of ovarian cancer plasticity. Am J Pathol.

[33] Rosanò, Varmi M, Salani D, Di Castro V, Spinella F, Natali PG, Bagnato A (2001). Endothelin-1 induces tumor proteinase activation and invasiveness of ovarian carcinoma cells. Cancer Res.

[34] Rosanò, Spinella F, Di Castro V, Dedhar S, Nicotra MR, Natali PG, Bagnato A (2006). Integrin-linked kinase functions as a downstream mediator of endothelin-1 to promote invasive behavior in ovarian carcinoma. Mol Cancer Ther.

[35] Salani D, Di Castro V, Nicotra MR, Rosanò, Tecce R, Venuti A, Natali PG, Bagnato A (2000). Role of endothelin-1 in neovascularization of ovarian carcinoma. Am J Pathol.

[36] Bagnato A, Salani D, Di Castro V, Wu-Wong JR, Tecce R, Nicotra MR, Venuti A, Natali PG (1999). Expression of endothelin 1 and endothelin A receptor in ovarian carcinoma: evidence for an autocrine role in tumor growth. Cancer Res.

[37] Nagaraj AB, Joseph P, Kovalenko O, Singh S, Armstrong A, Redline R, Resnick K, Zanotti K, Waggoner S, DiFeo A (2015). Critical role of Wnt/β-catenin signaling in driving epithelial ovarian cancer platinum resistance. Oncotarget.

[38] Rodriguez A, Griffiths-Jones S, Ashurst JL, Bradley A (2004). Identification of mammalian microRNA host genes and transcription units. Genome Res.

[39] Baffa R, Fassan M, Volinia S, O'Hara B, Liu CG, Palazzo JP, Gardiman M, Rugge M, Gomella LG, Croce CM, Rosenberg A (2009). MicroRNA expression profiling of human metastatic cancers identifies cancer gene targets. J Pathol.

[40] Boufraqech M, Nilubol N, Zhang L, Gara SK, Sadowski SM, Mehta A, He M, Davis S, Dreiling J, Copland JA, Smallridge RC, Quezado MM, Kebebew E (2015). miR30a inhibits LOX expression and anaplastic thyroid cancer progression. Cancer Res.

[41] Cheng CW, Wang HW, Chang CW, Chu HW, Chen CY, Yu JC, Chao JI, Liu HF, Ding SL, Shen CY (2012). MicroRNA-30a inhibits cell migration and invasion by downregulating vimentin expression and is a potential prognostic marker in breast cancer. Breast Cancer Res Treat.

[42] Qi F, He T, Jia L, Song N, Guo L, Ma X, Wang C, Xu M, Fu Y, Li L, Luo Y (2015). The miR-30 family inhibits pulmonary vascular hypermeability in the premetastatic phase by direct targeting of Skp2 in mice. Clin Cancer Res.

[43] Schmittgen TD, Livak KJ (2008). Analyzing real-time PCR data by the comparative C(T) method. Nat Protoc.

[44] Storey JD (2002). A direct approach to false discovery rates. J. R. Statist. Soc. B.

[45] Kaplan FL, Meier P (1958). Non parametric estimation from incomplete observations. Am J Stat Assoc.

[46] Mantel N (1966). Evaluation of survival data and two new rank order statistics arising in its consideration. Cancer Chemother Rep.

[47] Cox DR (1972). Regression models and life tables. J R Stat Soc.

