# Supplementary material for: miR-30a inhibits endothelin A receptor and chemoresistance in ovarian carcinoma
Source: Oncotarget. 2015 Dec 10;7(4):4009–23. doi: 10.18632/oncotarget.6546 (PMC4826186; doi:10.18632/oncotarget.6546)
Supplement: Supplementary file 1 [file oncotarget-07-4009-s001.pdf]

SUPPLEMENTARY FIGURES AND TABLES

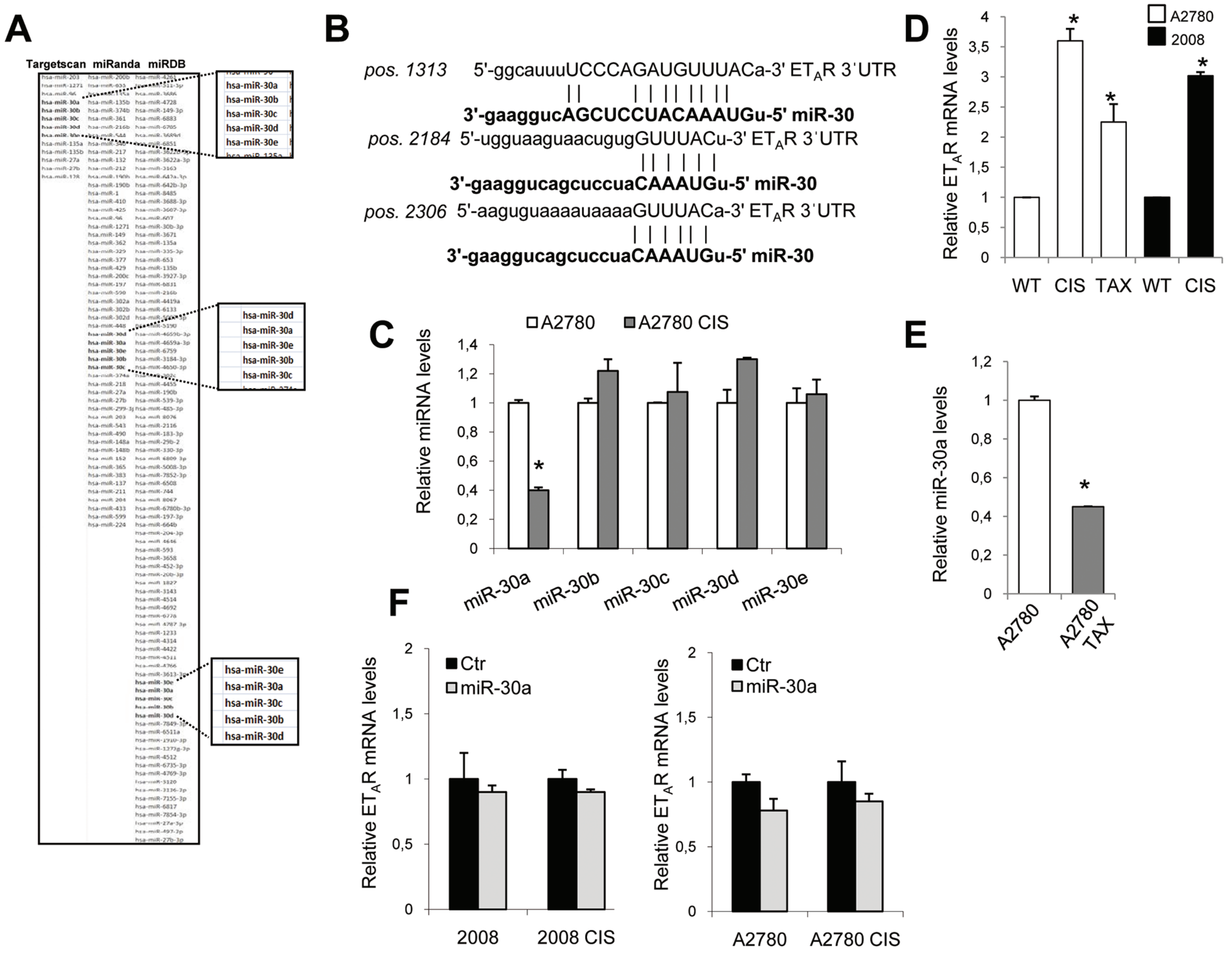

Supplementary Figure S1: miR-30a is downregulated in chemoresistant EOC cells. A. Putative miRNA able to bind the ET_AR 3′UTR predicted with the bioinformatic platforms TargetScan, miRanda and miRDB. **B.** Sequence alignment of miR-30 family with the seed binding sequences of the 3′UTR region of ET_AR mRNA. **C.** Expression of miR-30 family members in sensitive and resistant A2780 cells measured by qPCR. miRNA levels are normalized using endogenous U6 snRNA. Values are the mean ± SD ($n = 3$; *, $p < 0.005$ compared to sensitive cells). **D.** Expression of ET_AR mRNA in sensitive and resistant A2780 and 2008 cell lines measured by qPCR. ET_AR levels were normalized to cyclophilin-A. Values are the mean ± SD ($n = 3$; *, $p < 0.005$ compared to sensitive cells). **E.** Expression of miR-30a in sensitive and taxol-resistant A2780 cells measured by qPCR. miRNA levels are normalized using endogenous U6 snRNA. Values are the mean ± SD ($n = 3$; *, $p < 0.005$ compared to sensitive cells). **F.** ET_AR mRNA levels in 2008 and A2780 sensitive and resistant cells transfected with Ctr or mimic-miR-30a evaluated by qPCR. Values are the mean ± SD ($n = 3$).

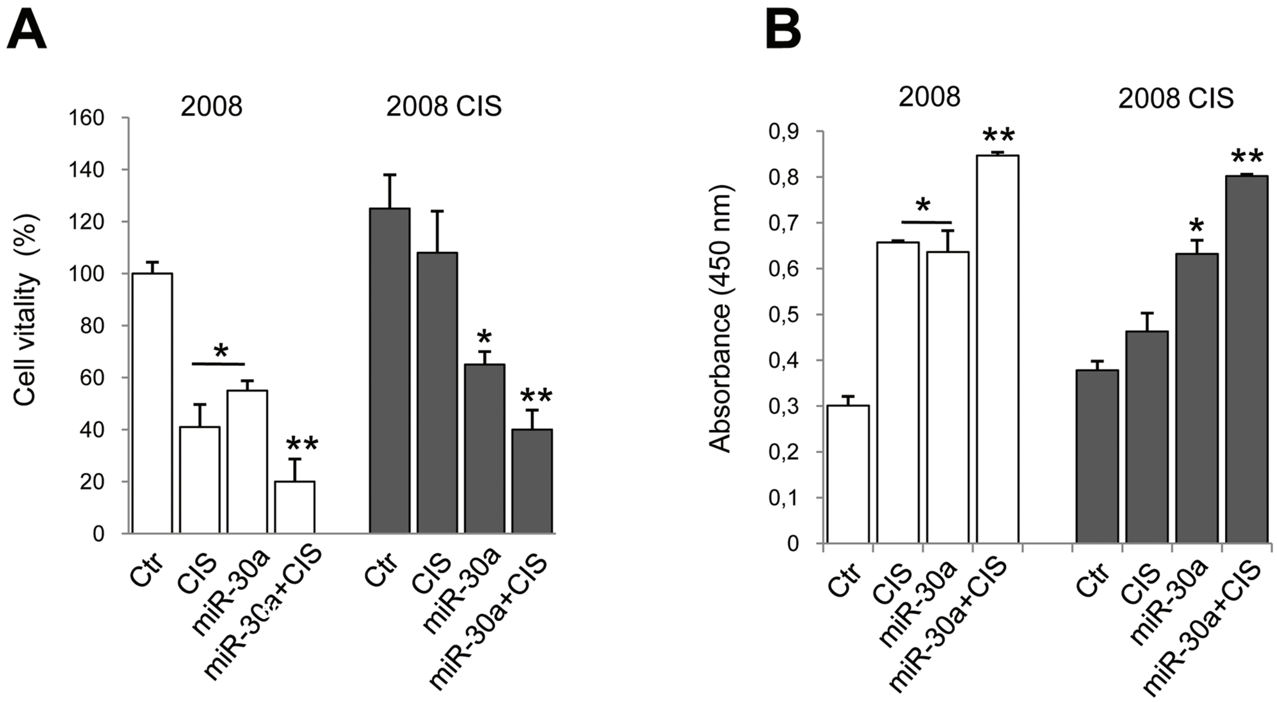

Supplementary Figure S2: Ectopic expression of miR-30a sensitizes EOC cells to cisplatinum-induced apoptosis. A. Cell viability of sensitive and resistant 2008 cells transfected with Ctr or mimic-miR-30a and treated with cisplatinum (CIS; 1 μM) for 72 h alone or in combination. Values are the mean ± SD ($n = 3$; *, $p < 0.05$ vs Ctr; **, $p < 0.05$ vs cisplatinum-treated cells). **B.** Detection of DNA fragmentation in sensitive and resistant 2008 cells cultured for 72 h as indicated. Values are the mean ± SD ($n = 3$; *, $p < 0.05$ vs Ctr; **, $p < 0.05$ vs cisplatinum-treated cells).

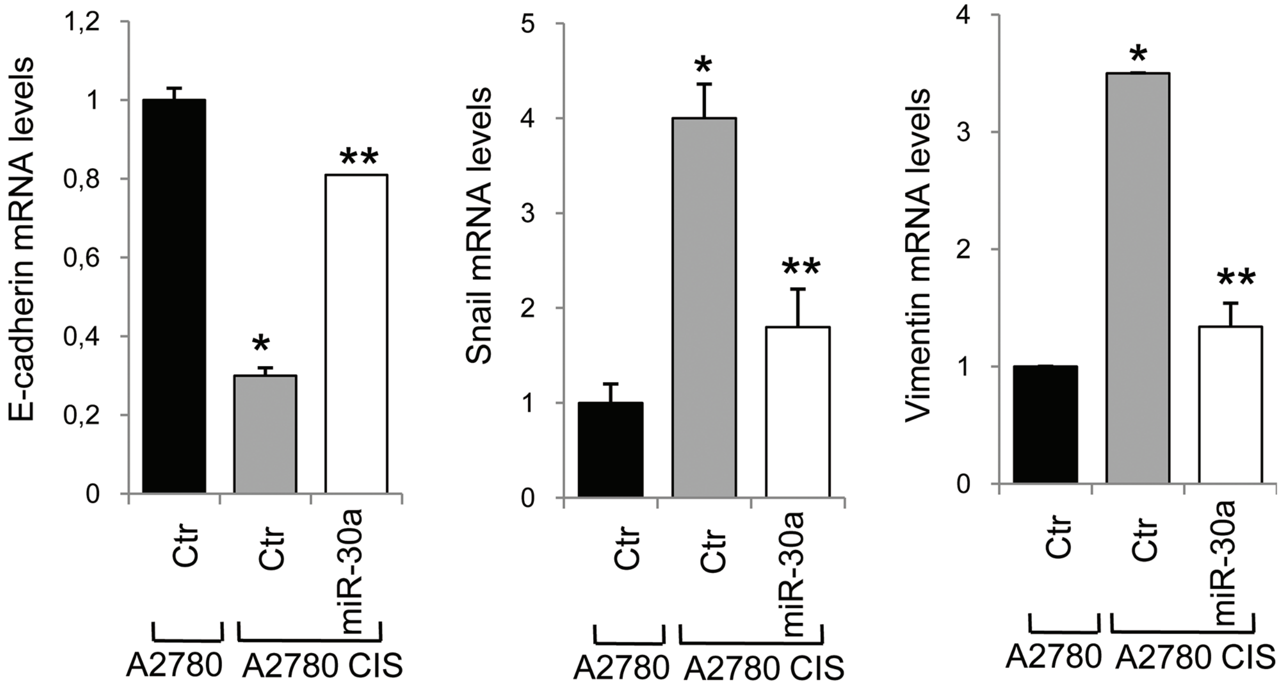

Supplementary Figure S3: miR-30a inhibits EMT phenotype. E-cadherin, Snail and vimentin mRNA expression in A2780 or A2780 CIS cells transfected with mimic-miR control (Ctr) or mimic-miR-30a evaluated by qPCR. Cyclophilin-A is used to normalize. Values are the mean ± SD ($n = 3$; *, $p < 0.05$ vs Ctr of sensitive cells; **, $p < 0.05$ vs Ctr of resistant cells).

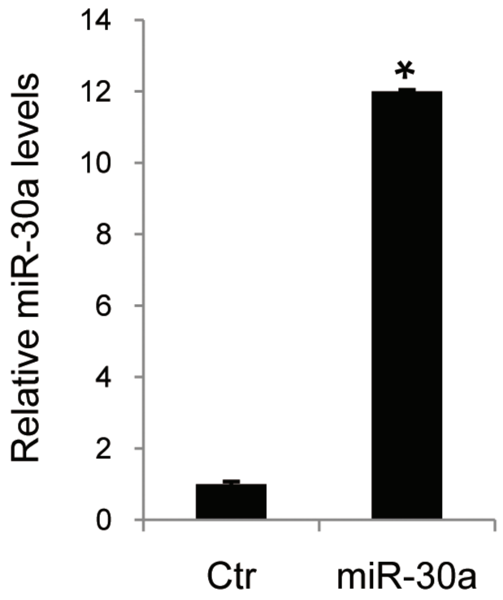

Supplementary Figure S4: miR-30a expression in stably transfected chemoresistant EOC cells. qPCR for miR-30a expression in 2008 CIS stably transfected with miR-30a or with vector control (Ctr), normalized using endogenous U6 snRNA. Values are the mean ± SD ($n = 3$; *, $p < 0.05$ compared to Ctr).

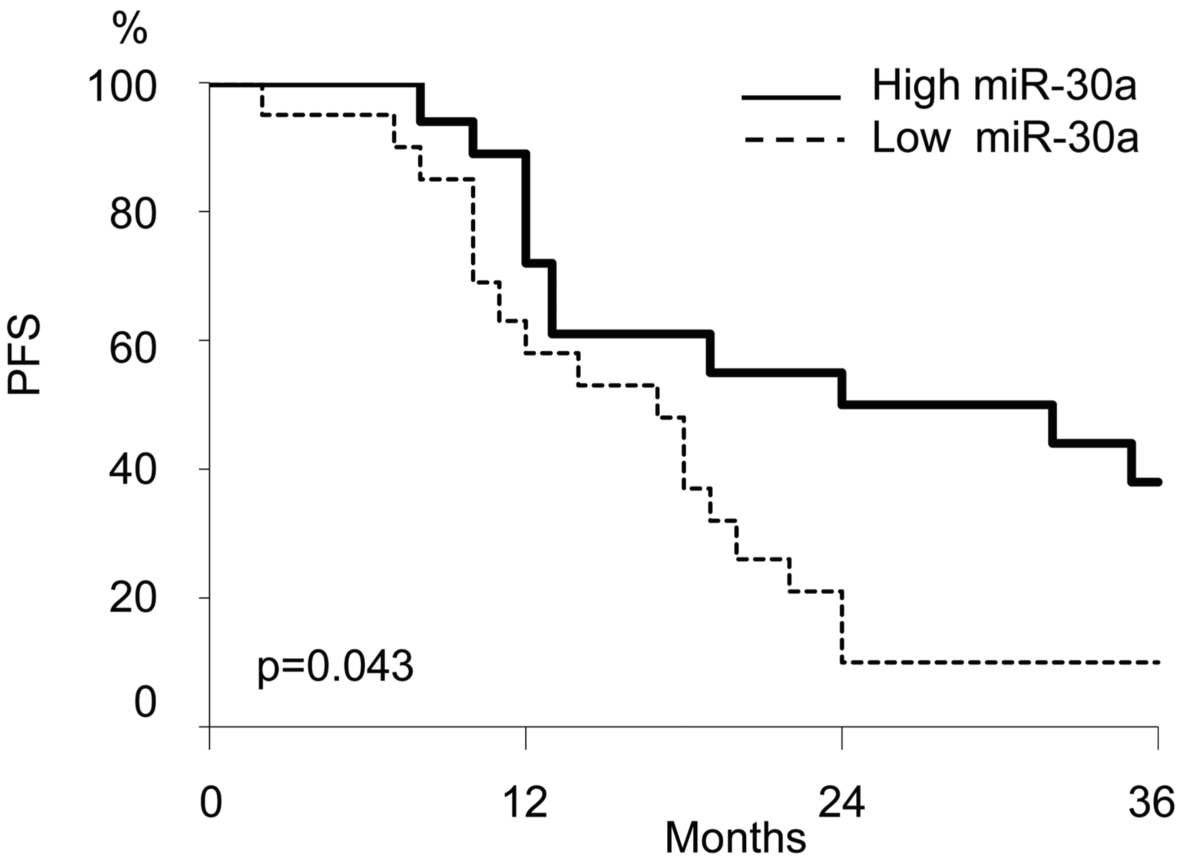

Supplementary Figure S5: miR-30a correlates with poor survival in EOC patients. Progression free survival (PFS) curves according to miR-30a expression in 39 EOC patients ($p = 0.043$).

Supplementary Table S1: Patient characteristics and miR-30a expression

Characteristics	All cases	miR-30a expression		
		Low	High	
	No.	No. (%)	No. (%)	p value[a]
**All**	39	22 (56.4)	17 (43.6)	
**Age, yrs**				
≤65	31	18 (58.1)	13 (41.9)	0.7
> 65	8	4 (45.4)	4 (55.5)	
**Histotype**				
Serous	28	17 (60.7)	11 (39.3)	
Other	11	5 (45.4)	6 (54.5)	0.5[b]
**Grade**				
G1–2	4	2 (50.0)	2 (50.0)	
G3	29	18 (62.1)	11 (37.9)	0.9
n.a.	6			
**Stage**				
II	6	3 (50.0)	3 (50.0)	
III-IV	33	19 (57.6)	14 (42.4)	0.9
**Ascites**				
No	13	6 (46.1)	7 (53.8)	
Yes	26	16 (61.5)	10 (30.5)	0.5

[a]calculated by Fisher's exact test for proportion
[b]calculated after grouping serous versus other histologic subtypes

Supplementary Table S2: Distribution of EOC patients according to ET_AR and miR-30a expressing tumors

ET_AR	miR-30a		
		Low	High
	No.	No. (%)	No. (%)
**Low**	13	4 (30.8)	9 (69.2)
**High**	26	18 (69.2)	8 (30.8)

p = 0.039 calculated by Fisher's exact test for proportion
